# Supplementary material for: The folate cycle enzyme MTHFD2 induces cancer immune evasion through PD-L1 up-regulation
Source: Nat Commun. 2021 Mar 29;12:1940. doi: 10.1038/s41467-021-22173-5 (PMC8007798; doi:10.1038/s41467-021-22173-5)
Supplement: Supplementary file 1 — Supplementary Information [file 41467_2021_22173_MOESM1_ESM.pdf]

Supplementary Figure 1

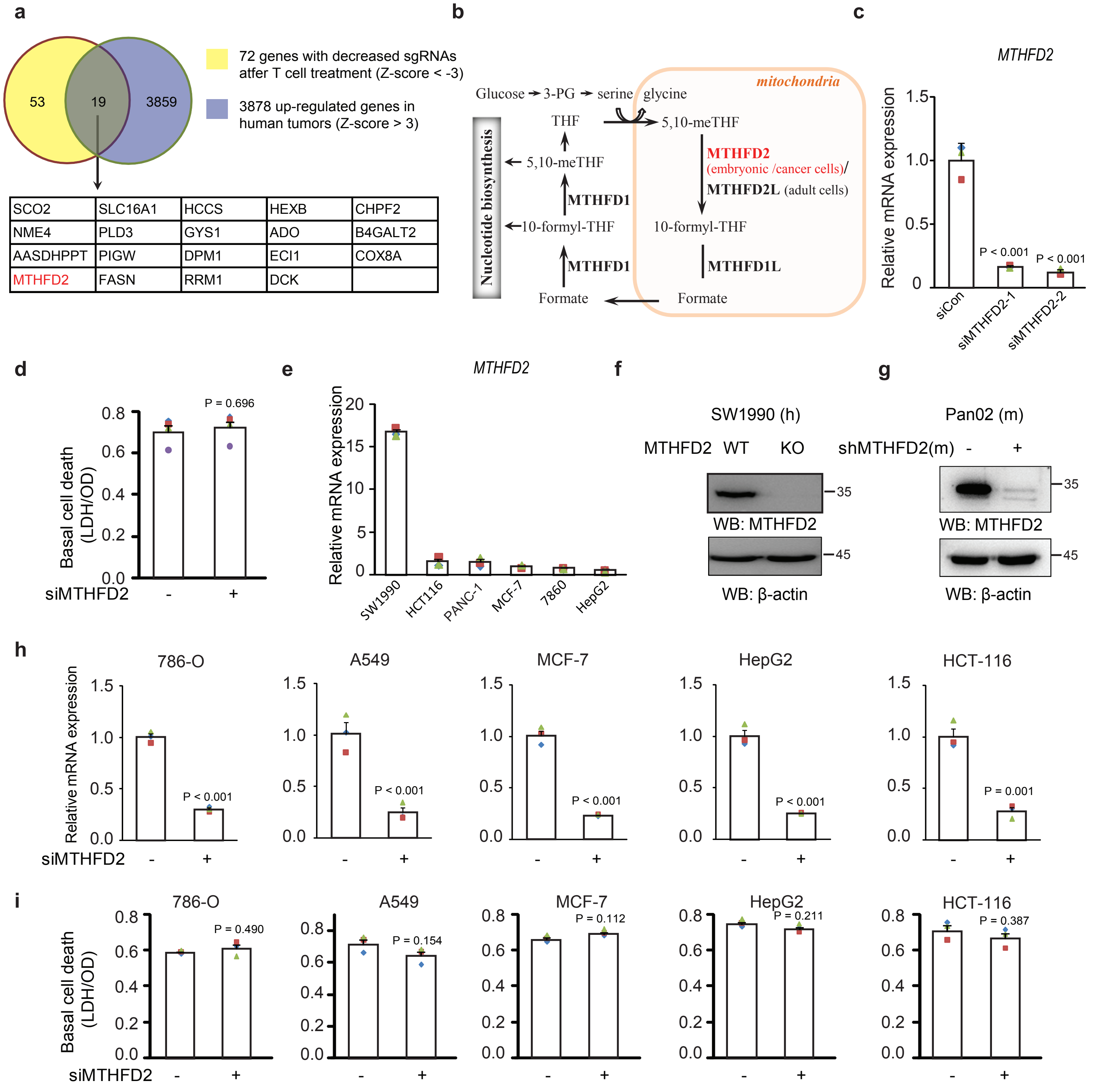

Supplementary Figure 1| Folate cycle enzymes contribute to tumour resistance against cytotoxic T cells. **a**, 72 genes were indentified that the sgRNAs of which decreased in T cell group (Z-score(T) <-3, P<0.1), 3878 over-expressed genes were selected in human tumors (Z-score＞3) from a previous meta-analysis (also see Supplementary Data 1 and 2). Among them, 19 genes up-regulated in tumours were selected and listed in the table. **b**, Schematic diagram of MTHFD2 in the mitochondria and cytoplasm. **c**, Relative mRNA levels of MTHFD2 in SW1990 with siRNAs were analyzed by real-time PCR. (n=3 independent experiments) **d**, LDH release in SW1990 with MTHFD2 siRNAs. (n=4 independent experiments) **e**, mRNA level of MTHFD2 in indicated cell lines were analyzed by real-time PCR. (n=3 independent experiments) **f-g**, MTHFD2 protein in SW1990 cells stably with indicated sgRNAs (**f**) and Pan02 cells with indicated shRNAs (**g**) were analyzed by immunoblotting. **h**, mRNA levels of MTHFD2 in indicated cells transfected with MTHFD2 siRNAs were analyzed by real-time PCR. (n=3 independent experiments) **i**, LDH release in indicated cells transfected with MTHFD2 siRNAs. (n=3 independent experiments) In c-e, h and i, the values are presented as mean ± s.e.m., P value represented (Student's t-test, two-sided) with control or the indicated groups.

Supplementary Figure 2

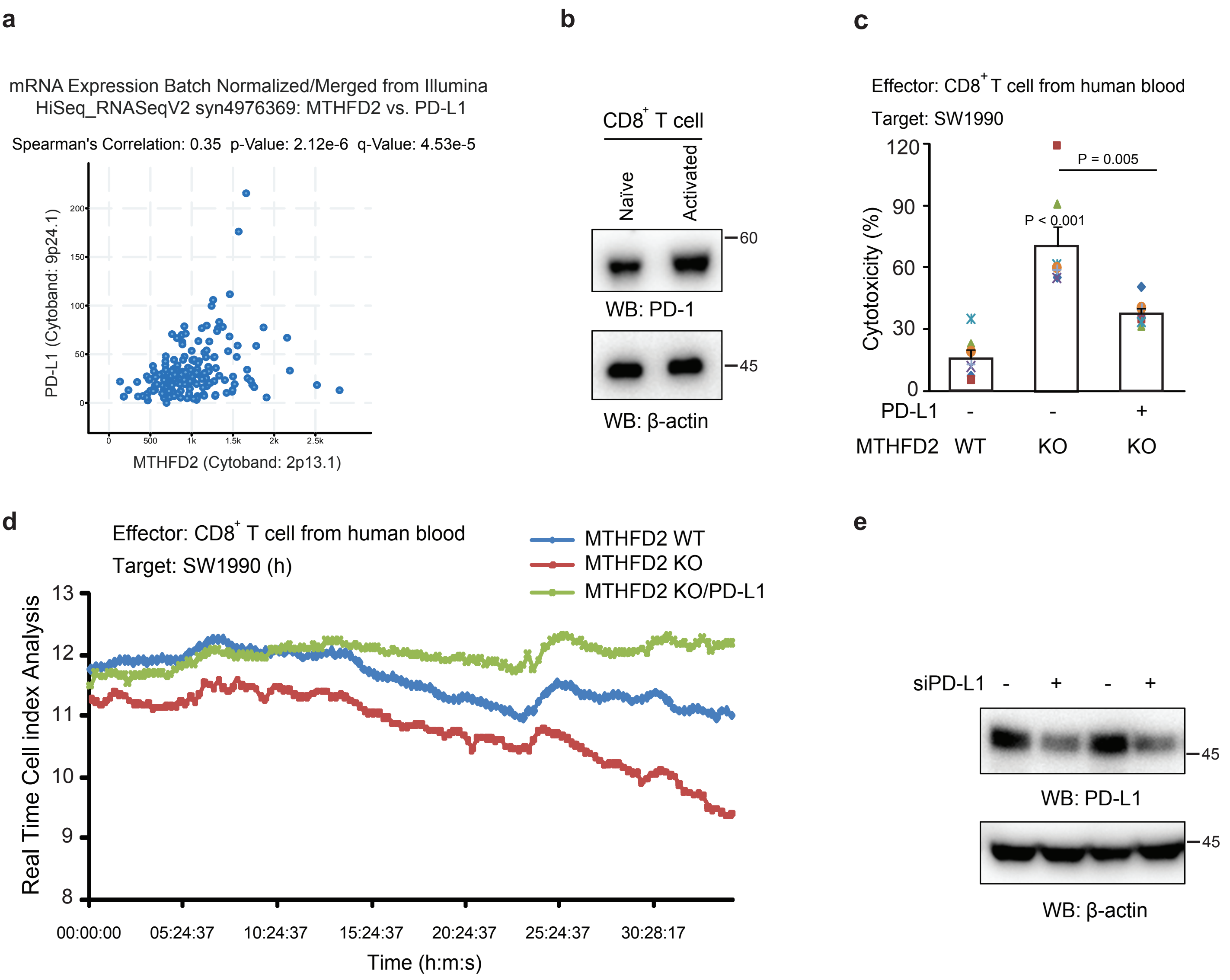

Supplementary Figure 2| MTHFD2 promotes tumour immune evasion via upregulating PD-L1 expression. a, Spearman's correlation between PD-L1 and MTHFD2 in Pancreatic cancer clinical samples from TCGA database. **b**, PD-1 expression were detected by immunoblotting in CD8+ T cells activated by 2 μg/ml CD3, CD28 and 10 ng/ml IL-2 for 24 hours. **c**, SW1990 cells wildtype or MTHFD2 KO cells expressing PD-L1, were incubated with activated human CD8+ effector T cells for 16 hours and the cytotoxicity were measured by LDH release assay. (n=7 independent experiments) **d**, Real-time Cell index analysis in WT or MTHFD2-KO SW1990 cells transfected with indicated expressing vectors, were incubated with activated human CD8+ T-cells in xCELLigence Plates. **e**, PD-L1 protein were analyzed by immunoblotting in SW1990 cells transfected with PD-L1 siRNA. In c, the values are presented as mean ± s.e.m., P value represented (Student's t-test, two-sided) with control or the indicated groups.

Supplementary Figure 3

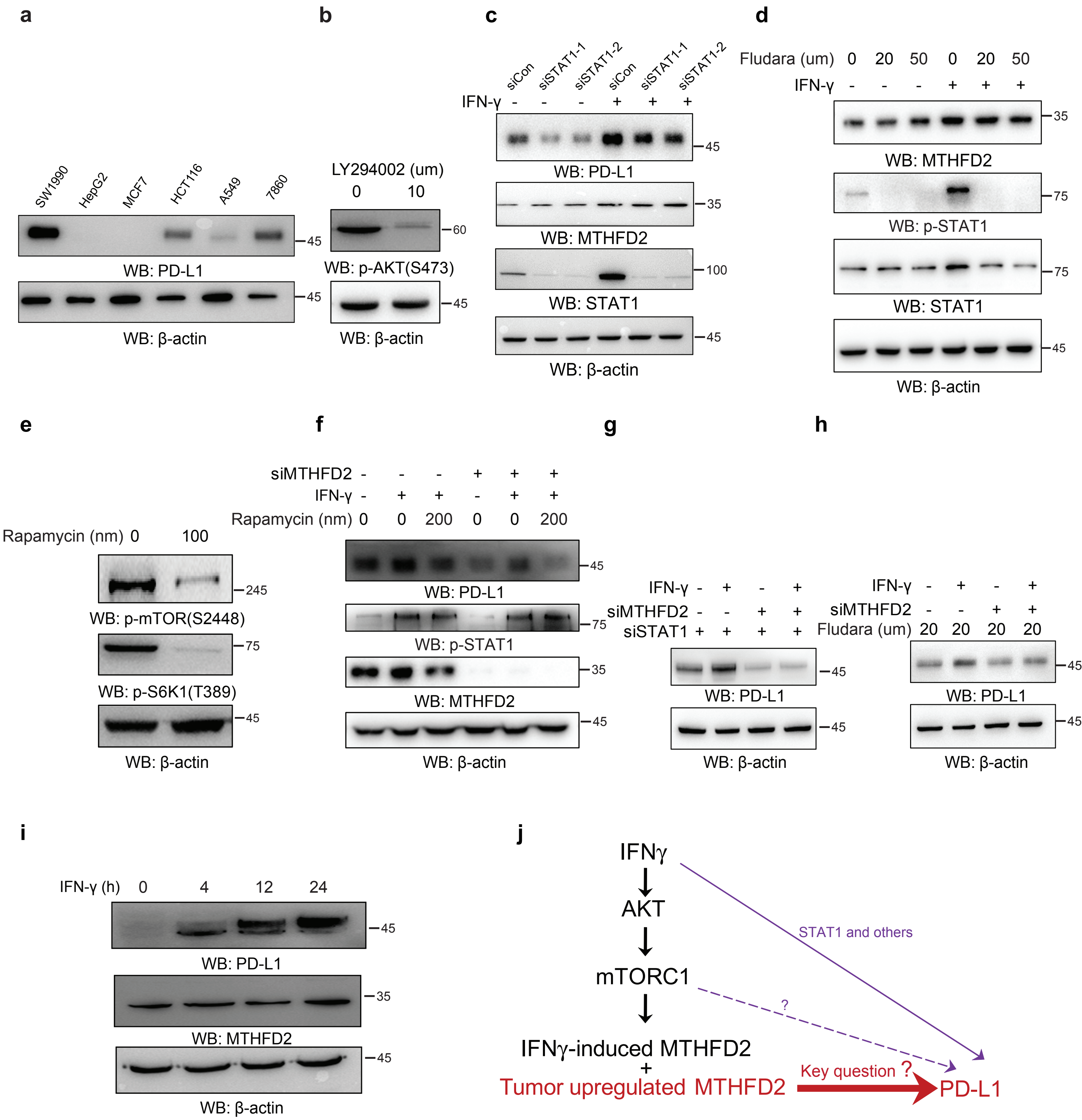

Supplementary Figure 3| MTHFD2 is induced by IFN-γ and involved in IFN-γ-mediated PD-L1 regulation. **a,** PD-L1 protein in indicated cells were analyzed by immunoblotting. **b**, p-AKT(S473) were analysed in SW1990 treated with AKT inhibitor LY294002 10 μM for 24 hours. **c-d**, SW1990 cells transfected with indicated siRNAs for 24 hours and then treated with 20 ng/ml IFN-γ for another 24 hours (**c**), or 20, 50 μM Fludara with 20 ng/ml IFN-γ for 24 hours (**d**), immunoblotting analyses were performed using the indicated antibodies. **e**, p-mTOR(S2448) and p-S6K1(T389) were analysed in SW1990 treated with rapamycin 100 nM for 24 hours. **f-h**, SW1990 cells transfected with indicated siRNAs for 24 hours and then treated with 20 ng/ml IFN-γ (**f-h**), 100nM rapamycin (**f**) or 20 uM Fludara (**h**) for another 24 hours, immunoblotting analyses were performed using the indicated antibodies. **i**, SW1990 cells treated with 20 ng/ml IFN-γ for indicated times, immunoblotting analyses were performed using the indicated antibodies. **j**, A model showing the regulatory relationships among IFN-γ, MTHFD2 and PD-L1 with known and unknown mechanisms.

Supplementary Figure 4

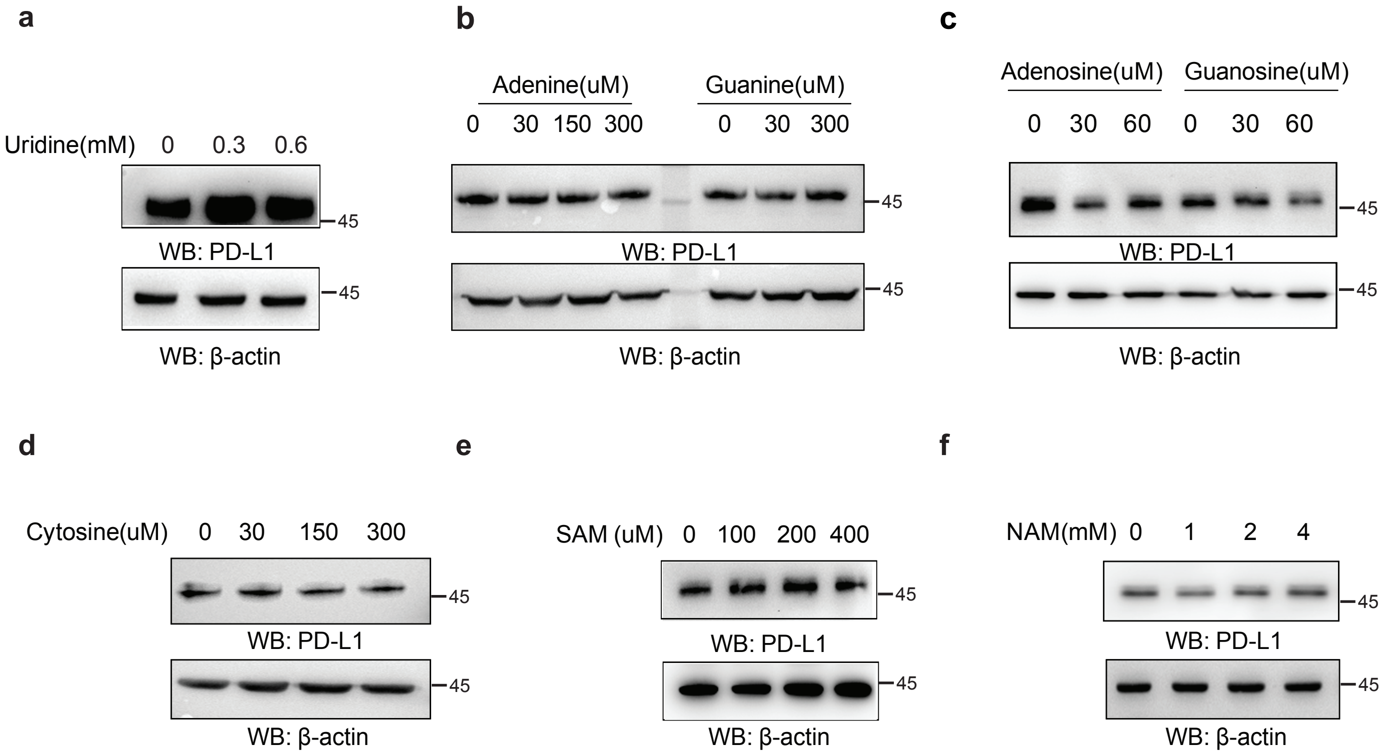

Supplementary Figure 4| Metabolic function of MTHFD2 promotes protein PD-L1 transcription through uridine-related-metabolites. a-f, SW1990 cells were treated with indicated concentration of indicated metabolites for 24 hours, immunoblotting analyses were performed using the indicated antibodies. SAM for S-adenosylmethionine, NAM for nicotinamide.

Supplementary Figure 5

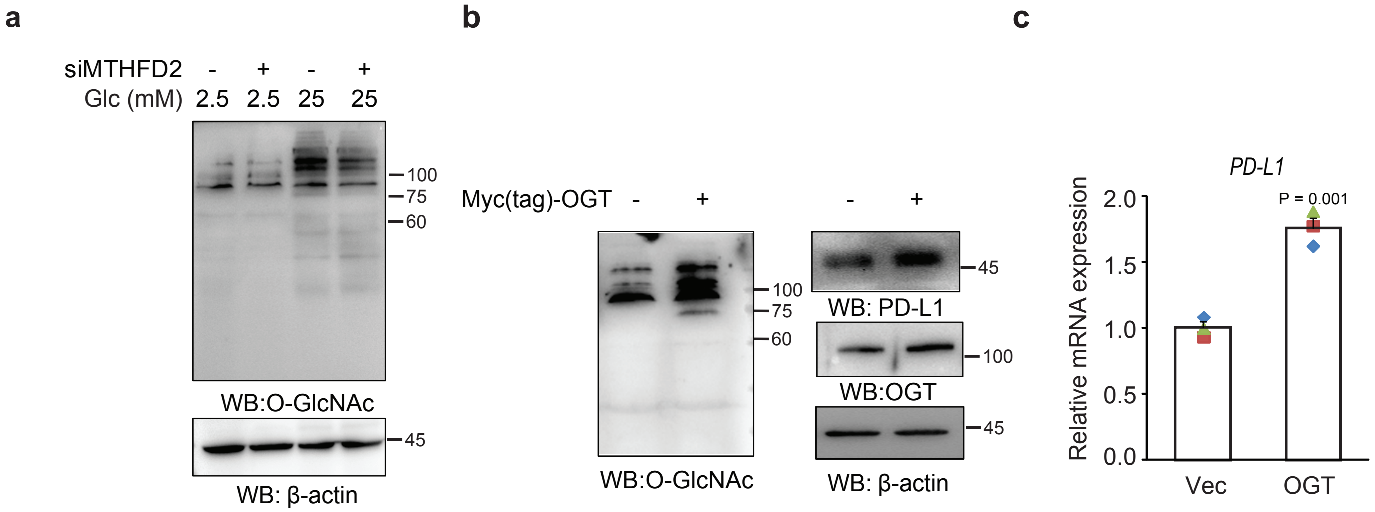

Supplementary Figure 5| Protein O-GlcNAcylation mediates MTHFD2 and uridine-related-metabolites enhanced PD-L1 transcription. a-b, SW1990 cells transfected with indicated siRNAs or indicated expressing vectors, or treated by 2.5, 25 mM glucose for 24 hours, immunoblotting analyses were performed using the indicated antibodies. **c**, SW1990 cells transfected with indicated expressing vectors, PD-L1 mRNA levels were analyzed by real-time PCR. (n=3 independent experiments) In c, the values are presented as mean ± s.e.m., **represents p<0.01 (Student’s t-test, two-sided) with vec group.

Supplementary Figure 6

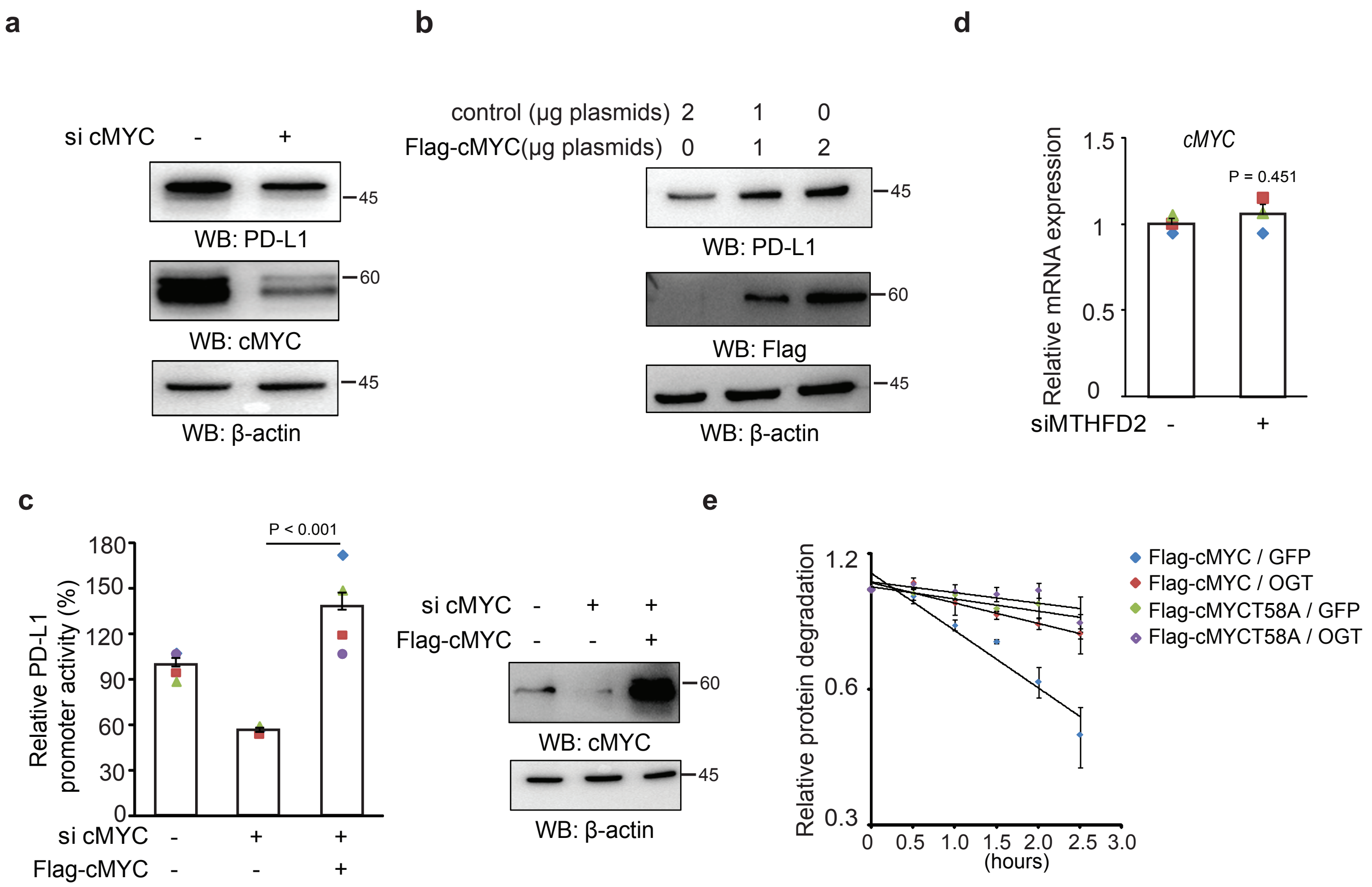

Supplementary Figure 6| MTHFD2 promotes PD-L1 transcription through cMYC O-GlcNAcylation. a-b, SW1990 cells transfected with indicated siRNAs or indicated expressing vectors, immunoblotting analyses were performed using the indicated antibodies. **c**, SW1990 cells transfected with indicated expressing vectors, the transcriptional activity of PD-L1 promoter (from-2000 bp to 0 bp) is examined by a luciferase-based reporter assay (n=5 independent experiments); immunoblotting analyses were performed using the indicated antibodies. **d**, SW1990 cells transfected with MTHFD2 siRNA, cMYC mRNA levels were analyzed by real-time PCR. (n=3 independent experiments) **e**, Analysis of the apparent half-life time of wild-type and mutant cMYC in cycloheximide treated cells transfected with indicated vectors. (n=3 independent experiments) In c-e, the values are presented as mean ± s.e.m., P value represented (Student's t-test, two-sided) with control or the indicated groups.

Supplementary Figure 7

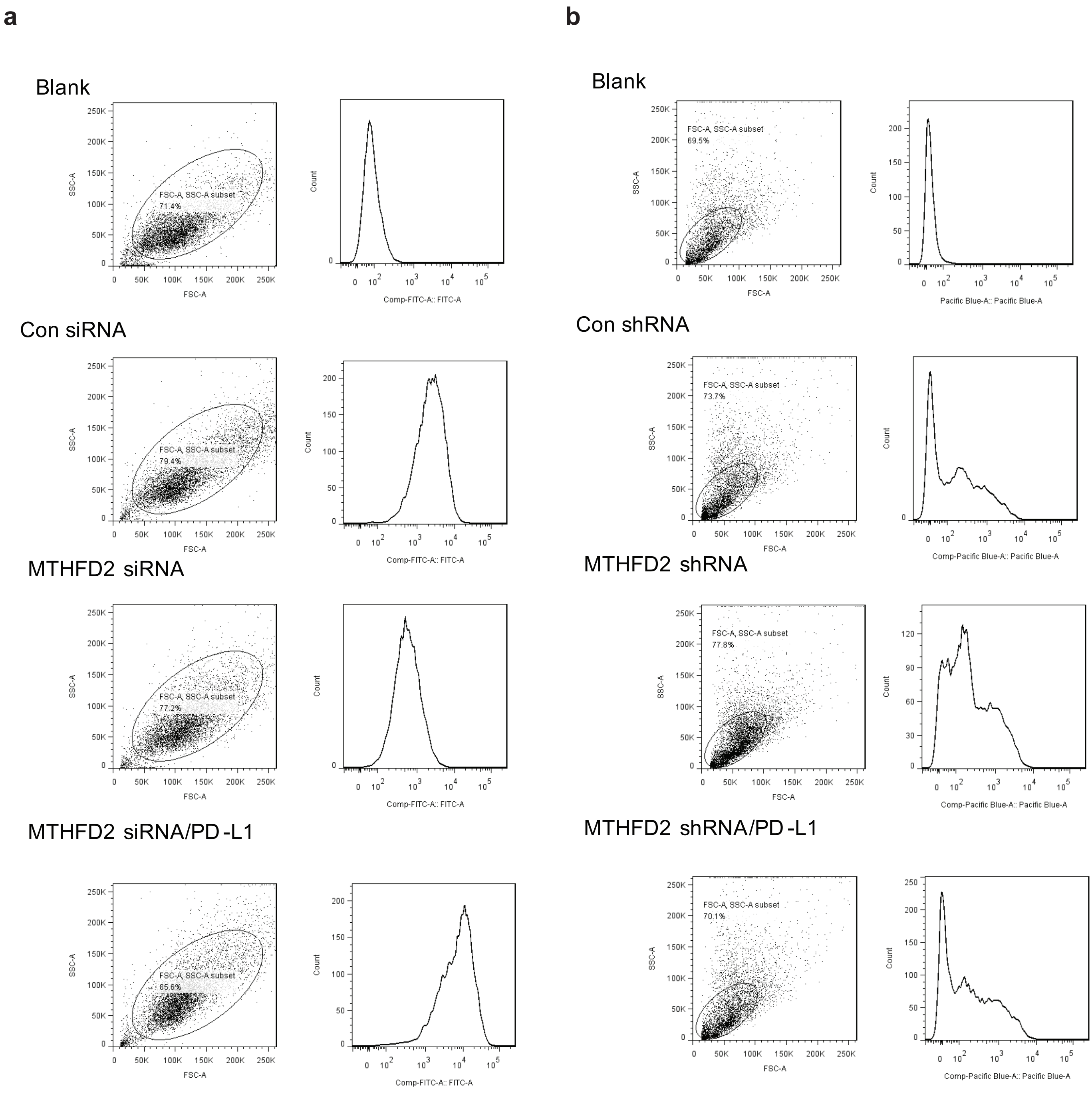

Supplementary Figure 7| Flow cytometry analysis of SW1990 cells in main Fig 2f and tumor tissues in C57 mice in main Fig 7f. a left, Gating based on FSC-A and SSC-A of SW1990 cells with indicated treatment were shown. **a right**, The cell-membrane localized PD-L1 in SW1990 cells with indicated treatment were shown in histogram. **b left**, Cells digested from indicate tumour tissues in C57 mice were gated based on FSC-A and SSC-A. **b right**, Cells digested from indicate tumour tissues in C57 mice were stained with anti-CD45 antibody and displayed in histogram.
