# Supplementary material for: The folate cycle enzyme MTHFD2 induces cancer immune evasion through PD-L1 up-regulation
Source: Nat Commun. 2021 Mar 29;12:1940. doi: 10.1038/s41467-021-22173-5 (PMC8007798; doi:10.1038/s41467-021-22173-5)
Supplement: Supplementary file 3 — Description of Additional Supplementary Files [file 41467_2021_22173_MOESM3_ESM.pdf]

Description of Additional Supplementary Files

Supplementary Data 1| The analysis of metabolic CRISPR library screen by MAGeCK

Supplementary Data 2| Up-regulated genes in human tumors (cited data)

Supplementary Data 3| Altered genes in siMTHFD2 cancer cells in RNA-seq

Supplementary Data 4| MTHFD2 correlated genes in TCGA clinical samples (RNASeqV2 syn4976369TGCA)

Supplementary Data 5| Metabolomics analysis of siMTHFD2 cancer cells by MS

Supplementary Data 6| List of primers used
