# Supplementary material for: The folate cycle enzyme MTHFD2 induces cancer immune evasion through PD-L1 up-regulation
Source: Nat Commun. 2021 Mar 29;12:1940. doi: 10.1038/s41467-021-22173-5 (PMC8007798; doi:10.1038/s41467-021-22173-5)
Supplement: Supplementary file 9 — Supplementary Data 6 [file 41467_2021_22173_MOESM9_ESM.docx]

| cloning primers | sequence (5'-3') |
| --- | --- |
| sgMTHFD2_1F | CACCGCTCGCGGCAGTTCGGTAAGA |
| sgMTHFD2_1R | AAACTCTTACCGAACTGCCGCGAGC |
| sgMTHFD2_2F | CACCGGTGTGGCCGTTTGTTGCCTG |
| sgMTHFD2_2R | AAACCAGGCAACAAACGGCCACACC |
| sgMTHFD2_3F | CACCGGCTGCAGGTAAGAACACAAG |
| sgMTHFD2_3R | AAACCTTGTGTTCTTACCTGCAGCC |
| MTHFD2_D168E_F | cggtaacatggaatactgctccaaacacattcgtcct |
| MTHFD2_D168E_R | aggacgaatgtgtttggagcagtattccatgttaccg |
| human PD-L1_F | ATGAGGATATTTGCTGTCTTTATATTCATGACCTACTG |
| human PD-L1_R | CGTCTCCTCCAAATGTGTATCACTTTGC |
| mouse PD-L1_F | ATGAGGATATTTGCTGGCATTATATTCACAGC |
| mouse PD-L1_R | CGTCTCCTCGAATTGTGTATCATTTCGG |
| OGT shRNA_F | TTTATCAGGATTGTGCATG |
| OGT shRNA_R | AAATTGATATAAGCATCCA |
| MYC-T58A _F | AGCTGCTGCCCGCCCCGCCCCTG |
| MYC-T58A _R | CAGGGGCGGGGCGGGCAGCAGCT |
| lentiCRISPR F1 | AATGGACTATCATATGCTTACCGTAACTTGAAAGTATTTCG |
| lentiCRISPR R1 | CTTTAGTTTGTATGTCTGTTGCTATTATGTCTACTATTCTTTCC |

| qPCR primers | sequence (5'-3') |
| --- | --- |
| MTHFD2 _F | tggctgcgacttctctaatg |
| MTHFD2 _R | ccttccagaaatgacaacagc |
| MYC_F | GGCTCCTGGCAAAAGGTCA |
| MYC_R | CTGCGTAGTTGTGCTGATGT |
| PD-L1_F | TGGCATTTGCTGAACGCATTT |
| PD-L1_R | TGCAGCCAGGTCTAATTGTTTT |
| β-actin_F | cgtaccactggcatcgtgat |
| β-actin_R | aggtagtcagtcaggtcccg |
