# Supplementary material for: The folate cycle enzyme MTHFD2 induces cancer immune evasion through PD-L1 up-regulation
Source: Nat Commun. 2021 Mar 29;12:1940. doi: 10.1038/s41467-021-22173-5 (PMC8007798; doi:10.1038/s41467-021-22173-5)
Supplement: Supplementary file 10 — Reporting Summary [file 41467_2021_22173_MOESM10_ESM.pdf]

nature research

Corresponding author(s): Ting Wang, Qiujing Yu

Last updated by author(s): Feb 27, 2021

Reporting Summary

Nature Research wishes to improve the reproducibility of the work that we publish. This form provides structure for consistency and transparency in reporting. For further information on Nature Research policies, see our Editorial Policies and the Editorial Policy Checklist.

Statistics

For all statistical analyses, confirm that the following items are present in the figure legend, table legend, main text, or Methods section.

n/a	Confirmed	
☐	☒	The exact sample size (n) for each experimental group/condition, given as a discrete number and unit of measurement
☐	☒	A statement on whether measurements were taken from distinct samples or whether the same sample was measured repeatedly
☐	☒	The statistical test(s) used AND whether they are one- or two-sided<br>*Only common tests should be described solely by name; describe more complex techniques in the Methods section.*
☐	☒	A description of all covariates tested
☐	☒	A description of any assumptions or corrections, such as tests of normality and adjustment for multiple comparisons
☐	☒	A full description of the statistical parameters including central tendency (e.g. means) or other basic estimates (e.g. regression coefficient) AND variation (e.g. standard deviation) or associated estimates of uncertainty (e.g. confidence intervals)
☐	☒	For null hypothesis testing, the test statistic (e.g. F, t, r) with confidence intervals, effect sizes, degrees of freedom and P value noted<br>*Give P values as exact values whenever suitable.*
☒	☐	For Bayesian analysis, information on the choice of priors and Markov chain Monte Carlo settings
☒	☐	For hierarchical and complex designs, identification of the appropriate level for tests and full reporting of outcomes
☐	☒	Estimates of effect sizes (e.g. Cohen's d, Pearson's r), indicating how they were calculated

Our web collection on statistics for biologists contains articles on many of the points above.

Software and code

Policy information about availability of computer code

Data collection	RTCA software 2.0 was used to collect the real-time cell data by xCELLigence plates.<br>Cells were acquired in a flow cytometer (BD FACS Verse) .<br>RNA sequencing on the RNAref+RNAseq Illumina platform were performed by BGI Genomics (BGISEQ-500).<br>Metabolomics analyses were performed using a UHPLC (1290 Infinity LC, Agilent Technologies) coupled to a quadrupole time-of-flight (AB SciexTripleTOF6600).<br>Cells were photoed by ZEN through the confocal microscope (Zeiss LSM800).<br>Paraffin sections were photoed by NIS-Elements through the NIKON upright microscope (Eclipse Ni-U).
Data analysis	RTCA software 2.0 was used to perform the real-time cell analysis by xCELLigence plates.<br>Flow cytometric data were analyzed using FlowJo 7.6.1. software (Tree Star).<br>CRISPR sgRNA (gDNA) sequencing results were analyzed by MAGeCK.<br>RNA sequencing results were analyzed by BGI Genomics.<br>Gene Set Enrichment in the MSigDB database were analyzed by GSEA v4.1.0.<br>The raw MS data (wiff.scan files) were converted to MzXML files using ProteoWizard MSConvert before importing into freely available XCMS software.<br>The Kaplan-Meier (KM) survival analysis was performed by comparing the survival data from the databases include GEO, EGA, and TCGA as described.

For manuscripts utilizing custom algorithms or software that are central to the research but not yet described in published literature, software must be made available to editors and reviewers. We strongly encourage code deposition in a community repository (e.g. GitHub). See the Nature Research guidelines for submitting code & software for further information.

Data

Policy information about availability of data

All manuscripts must include a data availability statement. This statement should provide the following information, where applicable:

- Accession codes, unique identifiers, or web links for publicly available datasets
- A list of figures that have associated raw data
- A description of any restrictions on data availability

Source data are provided with this paper. Specifically, the source data for Figures 1c-e, 1g, 2c-i, 2k-n, 3a-e, 4a-b, 4d-i, 5b-g, 6b-h, 7a-d, and Supplementary Figures 1c-i, 2b-c, 2e, 3a-i, 4a-f, 5a-c, 6a-d have been provided as Source Data File (an excel file contains 77 sheets).
The source information for other Figures and Supplementary Figures have also been provided as below:
The source information for Figures 1a, 1b and Supplementary Figure 1a have been provided in Supplementary Data 1 (an excel file contains 4 sheets) and Supplementary Data 2 (an excel file), and the related gDNA-seq data have been deposited in NCBI database with accession code PRJNA670077 [https://www.ncbi.nlm.nih.gov/bioproject/PRJNA670077/].
The source information for Figures 2a and 2b have been provided in Supplementary Data 3 (an excel file) and Supplementary Data 4 (an excel file), and the related RNA-seq data have been deposited in NCBI database with accession code PRJNA670286 [https://www.ncbi.nlm.nih.gov/bioproject/PRJNA670286/].
The source information for Figures 4c and 5a have been provided in Supplementary Data 5 (an excel file contains 3 sheets), and the related mass spectrometry metabolomic data have been deposited to the ProteomeXchange Consortium via the iProX partner repository with the dataset identifier PXD024228 [http://proteomecentral.proteomexchange.org/cgi/GetDataset?ID=PXD024228].
A complete list of all primers used has been supplied as a Supplementary Data 6.
The data in Fig. 1f was obtained and analyzed in KM plot website [http://kmplot.com/analysis/index.php?p=service&cancer=pancancer_rnaseq].
The data in Fig. 2b and Supplementary Figure 2a and Supplementary Data 4 were obtained and analyzed in cbioportal website [http://www.cbioportal.org; more specific hyperlink has been provided in Supplementary Data 4].
Any other information supporting the findings of this study is available from the corresponding author on reasonable request.

Field-specific reporting

Please select the one below that is the best fit for your research. If you are not sure, read the appropriate sections before making your selection.

☒ Life sciences ☐ Behavioural & social sciences ☐ Ecological, evolutionary & environmental sciences

For a reference copy of the document with all sections, see nature.com/documents/nr-reporting-summary-flat.pdf

Life sciences study design

All studies must disclose on these points even when the disclosure is negative.

Sample size	Samples size for each experiment is indicated in the figures or corresponding figure legends. The sample size of sufficient statistical power in mice study was chosen based on previous experience (PMID 28628081).
Data exclusions	No samples or animals were excluded from the analyses.
Replication	The experiments were successfully repeated. Clear statements have been put into Methods section and Figure legends.
Randomization	The mice were randomly put into separate/groups cages for experiments.
Blinding	For mice studies, the experiments were performed in a blinded fashion when possible. Downstream analyses of mouse samples (IHC, RT-PCR, FACS and WB) were performed in a blinded fashion, which means that people performing the assays were not aware of the treatment groups until the data analyses were completed.

Reporting for specific materials, systems and methods

We require information from authors about some types of materials, experimental systems and methods used in many studies. Here, indicate whether each material, system or method listed is relevant to your study. If you are not sure if a list item applies to your research, read the appropriate section before selecting a response.

Materials & experimental systems

n/a	Involved in the study
	☒ Antibodies
	☒ Eukaryotic cell lines
☒	Palaeontology and archaeology
	☒ Animals and other organisms
	☒ Human research participants
☒	Clinical data
☒	Dual use research of concern

Methods

n/a	Involved in the study
☒	ChIP-seq
	☒ Flow cytometry
☒	MRI-based neuroimaging

Antibodies

Antibodies used

The following primary antibodies were used for Western blotting. They are listed as antigen first, followed by dilution, host,supplier, catalog number and clone/lot number as applicable.
1) Anti-MTHFD2, 1:1000, Rabbit, Cell Signaling Technology, #41377, clone D8W9U;
2) Anti-PD-L1, 1:1000, Rabbit, Cell Signaling Technology, #13684, clone E1L3N®;
3) Anti-O-GlcNAc, 1:1000, Mouse,Cell Signaling Technology, #9875, clone CTD110.6;
4) Anti-p-STAT-1 T701, 1:1000,Rabbit, Cell Signaling Technology, #9167, clone 58D6;
5) Anti-STAT-1, 1:1000, Rabbit,Cell Signaling Technology, #14994, clone D1K9Y;
6) Anti-OGT, 1:1000, Rabbit, Abcam, ab96718;
7) Anti-FLAG® M2 Magnetic Beads, Sigma, #M8823, clone M2;
8) Anti-β-Actin−Peroxidase antibody, 1:10000, Mouse, Sigma, #A3854, cloneAC-15;
9) Anti-Flag, 1:1000, Rabbit, Invitrogen, # PA1-984B;
10) Anti-cMYC, 1:1,000, Rabbit, Proteintech, #10828-1-AP.

The following primary antibodies were used for flow cytometry. They are listed as antigen first, followed by dilution, host,supplier, catalog number and clone/lot number as applicable.
1) Anti-PD-L1, 1:200, Rabbit, Abcam, #205921, clone [28-8];
2) Anti-CD45 eFluor 450, 1:100, Rat, eBioscience, #48-0452-82, clone RA3-6B2.

The following primary antibodies were used for immunohistochemistry. They are listed as antigen first, followed by dilution, host,supplier, catalog number and clone/lot number as applicable.
1) Anti-MTHFD2, 1:200, Rabbit, Proteintech, #12270-1-AP;
2) Anti-PD-L1, 1:200, Rabbit, Cell Signaling Technology, #13684, clone E1L3N®;
3) Anti-O-GlcNAc, 1:200, Mouse,Cell Signaling Technology, #9875, clone CTD110.6;
4) Anti-CD8a, 1:200, Mouse,eBioscience, #14-0808-82, clone4SM15.

The following primary antibodies were used for cell activation. They are listed as antigen first, followed by using concentration, host,supplier, catalog number and clone/lot number as applicable.
1) Anti-human-CD3, 2 μg/ml, Mouse, BioLegend, #300314, clone HIT3a;
2) Anti-human-CD28, 2 μg/ml, Mouse, BioLegend, #302914, clone CD28.2;
3) Anti-mouse-CD3, 2 μg/ml, Rat, BioLegend, #100207;
4) Anti-mouse-CD28, 2 μg/ml, Syrian Hamster, BioLegend, #102111, clone 37.51.

The following recombination proteins were used forcell activation. They are listed as source first, followed by using concentration, expressedhost, supplier, catalog number and clone/lot number as applicable.
1) Anti-human-IL2, 10ng/ml, insect cells, Biolegend, #589102;
2) Anti-human-IFN-γ, 20ng/ml, CHO Stable Cells, Sino Biological, #11725-HNAS;
3) Anti-mouse-IFN-γ, 20ng/ml,HEK293 Cells, Sino Biological, #50709-MNAH.

Validation

All antibodies were purchased from commercial companies, and validated by the data sheets of themanufacturer or citations listed below.
The following primary antibodies were used for Western blotting.
1) Anti-MTHFD2, validated with Western blot analysis of extracts from HeLa and 293 cells (https://www.cellsignal.com/products/primary-antibodies/mthfd2-d8w9u-rabbit-mab/41377);
2) Anti-PD-L1, validated with Western blot analysis of extracts from KARPAS-299, SUP-M2, and PC-3 cells (https://www.cellsignal.com/products/primary-antibodies/pd-l1-e1l3n-xp-rabbit-mab/13684);
3) Anti-O-GlcNAc, validated with Western blot analysis of extracts from HeLa cells, untreated (-) or treated with PUGNAc (+), an inhibitor of N-acetyl-β-D-glucosaminidase(https://www.cellsignal.com/products/primary-antibodies/o-glcnac-ctd110-6-mouse-mab/9875);
4) Anti-p-STAT-1 T701, validated withWestern blot analysis of extracts from HeLa cells untreated or treated with interferon-α (IFN-α) (https://www.cellsignal.com/products/primary-antibodies/phospho-stat1-tyr701-58d6-rabbit-mab/9167?country=USA);
5) Anti-STAT-1, validated withWestern blot analysis of extracts from A549 cells or STAT1 knock-out cells (https://www.cellsignal.com/products/primary-antibodies/stat1-d1k9y-rabbit-mab/14994);
6) Anti-OGT, validated with in SW1990 cells in Lei Y et al.Oncogenesis (2020), PMID: 32060258;

7) Anti-FLAG® M2 Magnetic Beads, validated with various cells in Aitor Garzia et. AlNature communications (2017), PMID 28685749;
8) Anti-β-Actin–Peroxidase antibody, validated withvarious cells inBiola M Javierre et. alMolecular cancer research (2011), PMID 21737484;
9) Anti-Flag, validated withWestern blot of recombinant protein containing the epitope tag sequence DYKDDDDK (https://www.thermofisher.com/cn/zh/antibody/product/DYKDDDDK-Tag-Antibody-Polyclonal/PA1-984B);
10) Anti-cMYC, validated withvarious lysates subjected to SDS PAGE followed by Western blot with 10828-1-AP (c-MYC antibody) at dilution of 1:2000 (https://www.ptglab.com/products/MYC-Antibody-10828-1-AP.htm).

The following primary antibodies were used for flow cytometry.
1) Anti-PD-L1, validated with TALEN constructs targeting exon4 of human PD-L1, transcript variant 1 (NM_014143.3) and complete knock out (K.O) confirmed by deep sequencing in clone L2-14. Cell surface staining is almost completely eliminated in the L2987 L2-14 KO cell line. (https://www.abcam.com/pd-l1-antibody-28-8-ab205921.html#lb);
2) Anti-CD45 eFluor 450, validated by flow cytometric analysis of mouse splenocytes (https://www.thermofisher.com/cn/zh/antibody/product/CD45R-B220-Antibody-clone-RA3-6B2-Monoclonal/48-0452-82).

The following primary antibodies were used for immunohistochemistry.
1) Anti-MTHFD2, validated with Immunohistochemical analysis of paraffin-embedded human colon cancer using 12270-1-AP (MTHFD2 antibody) at dilution of 1:50 (https://www.ptglab.com/Products/MTHFD2-Antibody-12270-1-AP.htm);
2) Anti-PD-L1, validated with immunohistochemical analysis of paraffin-embedded human non-small cell lung carcinoma (https://www.cellsignal.com/products/primary-antibodies/pd-l1-e1l3n-xp-rabbit-mab/13684);
3) Anti-O-GlcNAc, validated with the pancreas on 3-week-old GLUL WT and GLUL knockout (KO) mice in Alex J Bott, et. al. Cell Report (2019), PMID: 31665640;
4) Anti-CD8a, validated with immunohistochemistry of formalin-fixed paraffin embedded mouse spleen (https://www.thermofisher.com/cn/zh/antibody/product/CD8a-Antibody-clone-4SM15-Monoclonal/14-0808-82).

The following primary antibodies were used for cell activation.
1) Anti-human-CD3, validated in many research, Sedelies KA, et al. 2004. J. Biol. Chem. 279:26581. (Activ); Rivollier A, et al. 2004. Blood 104:4029. (Activ); Scharschmidt E, et al. 2004. Mol. Cell Biol. 24:3860. (Activ); Smeltz RB. 2007. J. Immunol. 178:4786. (Activ);
2) Anti-human-CD28, validated in many research,Marti F, et al. 2001. J. Immunol. 166:197. (Costim); Jeong SH, et al. 2004. J. Virol. 78:6995. (Costim); Rivollier A, et al. 2004. Blood 104:4029. (Costim); Scharschmidt E, et al. 2004. Mol. Cell Biol. 24:3860. (Costim);
3) Anti-mouse-CD3, validated in many research,Chattopadhyay K, et al. 2008. Proc. Natl. Acad. Sci. USA 105:635.; Byrne AM, et al. 2012. Clin. Exp. Allergy 42:550.; Clouthier DL, Watts TH. 2014. Cytokine Growth Factor Rev. 25:91.;
4) Anti-mouse-CD28, validated in many research,Zhang N and He Y-W, 2005. J. Exp. Med. 202:395. (Costim); Terrazas LI, et al. 2005. Intl. J. Parasitology. 35:1349. (Costim); Perchonock CE, et al. 2006. Mol Cell Biol. 26(16):6005. (Costim); Wang W, et al. 2007. J. Immunol. 178:4885. (Costim); Pua HH, et al. 2007. J. Exp. Med. 204:25. (Costim).

The following recombination proteins were used forcell activation.
1) Anti-human-IL2, validated in many research, Wingender G, et al. 2011. J. Exp. Med. 208:1151.;
2) Anti-human-IFN-γ, validated in many publications, Goshima N, et al. (2008) Nomura N Nat Methods. 5: 1011-7.; Thiel DJ, et al. (2000) Structure. 8 (9): 927-36.Schoenborn JR, et al. (2007) Adv Immunol. 96: 41-101.;
3) Anti-mouse- IFN-γ, validated in many publications, Naylor SL, et al. (1983) J Exp Med. 157 (3): 1020-7.; Schoenborn JR, et al. (2007) Adv Immunol. 96: 41-101.

Eukaryotic cell lines

Policy information about cell lines

Cell line source(s)	SW1990, HepG2, A549, MCF-7, HCT-116, 786-O and Pan02 cell lines were from ATCC. HPDE cell line was from kerafast.
Authentication	Cell lines purchased were frozen at early passage, thus did not require additional authentication.
Mycoplasma contamination	The cells are routinely tested for mycoplasma contamination. The cell line was negative for mycoplasma.
Commonly misidentified lines (See ICLAC register)	No.

Animals and other organisms

Policy information about studies involving animals; ARRIVE guidelines recommended for reporting animal research

Laboratory animals	Six-week-old male C57BL/6J and Balb/c nude mice were used for tumor xenografts study. All animals were kept under specific pathogen free (SPF) and temperature-controlled environment with 12 h light/12 h dark cycle, and free access to food and water. OT-I mice were generated from C57BL/6 which contain transgenic inserts for mouse Tcra-V2 and Tcrb-V5 genes, and express a transgene encoding a T cell receptor that specifically recognized SIINFEKL peptide bound to MHC-I H-2kb. 8-week-old male OT-I mice were gifted from the third military medical university.
Wild animals	No wild animals were used in this study.
Field-collected samples	No field-collected samples were used in this study.
Ethics oversight	All animal studies were performed under the guidelines of the Animal Care and Use Committee of Tianjin Medical University. All

animal experiments were approved by the Ethics Committee of Tianjin Medical University.

Note that full information on the approval of the study protocol must also be provided in the manuscript.

Human research participants

Policy information about studies involving human research participants

Population characteristics	When the samples were collected, the characteristics of the 73 pancreatic cancer patients were below: age range 47 - 77 years, median 61 years; 45 males and 28 females; clinical phase 1A (8), 1B (26), 2A (13) and 2B (26).
Recruitment	The formalin fixed paraffin embedded sections of 73 patients have been used in this study. Among the 73 patients, 11 patients also suffered from diabetes, which may cause a limited level of bias. Buffy coats of healthy donors were purchased from Tianjin Blood Center.
Ethics oversight	The use of human pancreas tumor specimens and the database was obtained from Shanghai East Hospital of Tongji University and approved by the Ethics Committee of the Shanghai East Hospital of Tongji University and patient consent was obtained. The blood samples of healthy donors were only used for the present research and approved by the Ethics Committee of Tianjin Blood Center.

Note that full information on the approval of the study protocol must also be provided in the manuscript.

Flow Cytometry

Plots

Confirm that:

- [x] The axis labels state the marker and fluorochrome used (e.g. CD4-FITC).
- [x] The axis scales are clearly visible. Include numbers along axes only for bottom left plot of group (a 'group' is an analysis of identical markers).
- [x] All plots are contour plots with outliers or pseudocolor plots.
- [x] A numerical value for number of cells or percentage (with statistics) is provided.

Methodology

Sample preparation	SW1990 cells were transfected with siMTHFD2 and PD-L1 plasmid. Cells were scratched from the culture dish and washed with PBS. Each sample was incubated with PD-L1 antibody (abcam, clone28-8) for 30 min on ice in dark. The immunofluorescence of PD-L1 in SW1990 was analyzed by flow cytometry.<br>Xenografts from C57BL/6J mice were removed and minced into 1 mm3 pieces in the solution of 0.2% collagenase IV at 37°C for 4 hours with mild shake. Cells dissociated from the tissues were sieved through a 40-µm mesh and diluted to 10^6 cells/mL in PBS. Samples were incubated with CD45 eFluor 450 (eBioscience) for 30 min in dark. Cells were acquired in a FACS Verse flow cytometer (BD).
Instrument	Cells were acquired in a FACS Verse flow cytometer (BD).
Software	Data were analyzed using FlowJo software (Tree Star).
Cell population abundance	Cell population was gated according to its accumulation and could account for 80% of the whole events.
Gating strategy	Boundaries between "positive" and "negative" staining cell populations were defined according to samples stained with isotypes of indicated antibodies.

- [x] Tick this box to confirm that a figure exemplifying the gating strategy is provided in the Supplementary Information.
